# Firearm Storage and the Perceived Risks and Value of Firearms: Differences Among Firearm Owners and Nonfirearm Owners With and Without a History of Suicidal Ideation

**DOI:** 10.1111/sltb.70026

**Published:** 2025-05-27

**Authors:** Michael D. Anestis, Allison E. Bond

**Affiliations:** ^1^ New Jersey Gun Violence Research Center Rutgers School of Public Health New Brunswick New Jersey USA

**Keywords:** firearms, gun violence, secure storage

## Abstract

**Objective:**

To identify firearm behaviors and perceptions that differentiate firearm owners with a history of suicidal thoughts from other firearm owners and from nonfirearm owners with a history of suicidal thoughts.

**Methods:**

A nationally representative sample (*n* = 8009) of adults residing in the United States completed a self‐report survey May 15–May 28, 2024.

**Results:**

Firearm owners with a history of suicidal thoughts were 45% likelier to store firearms loaded and unlocked and reported a stronger belief that home firearm access increases suicide risk. Some subgroups of firearm owners more heavily represented among those with a history of suicidal thoughts (e.g., women) were more likely to store firearms loaded and unlocked if they endorsed a history of suicidal thoughts.

**Conclusions:**

Firearm owners with a history of suicidal thoughts are more likely to recognize the link between firearm access and suicide risk, but nonetheless are more likely to store firearms unsecured. Identification of firearm owners with suicidal ideation remains difficult, but findings point toward subgroups and beliefs to target with messages aimed at promoting secure firearm storage.

## 1 Introduction

Firearms account for over 50% of suicide deaths in the United States (Centers for Disease Control and Prevention 2024), and the presence of a firearm in the home increases the risk for suicide for all household members (Anglemyer et al. 2014). The risk for suicide is increased further when a firearm is stored in an unsecured (loaded, unlocked) (Anestis et al. 2017). Identification of individuals at risk for firearm suicide is difficult, as those at risk often do not seek mental health treatment prior to death (Bond et al. 2022a). Therefore, public health efforts that seek to identify trends in those at risk for suicide and offer population‐level interventions are needed. To do this, research needs to identify factors that characterize firearm owners with suicidal ideation relative to those without such histories.

Research has shown that specific demographic characteristics—such as being male, white, older, living in rural areas (Miller et al. 2019), and holding more conservative political views (Pew Research Center 2014)—are associated with a higher likelihood of firearm ownership. Additionally, research indicates that firearm owners are not a homogenous group and there are multiple demographic subgroups (Bryan et al. 2022a; Bond et al. 2022b). Firearm owners who endorse suicidal ideation represent a unique subgroup, but research has yet to explore whether their demographic profile differs from firearm owners without suicidal ideation. Understanding these differences may offer valuable insights for identifying profiles of firearm owners at risk of suicide. For example, awareness of such demographic differences may inform efforts at messaging on secure firearm storage, providing insight into how best to target firearm owners thinking about suicide but not identified as such due to avoidance of mental health care and reluctance to discuss their thoughts with others.

While research has started to explore factors that influence firearm storage habits, it is limited in its assessment of differences between those with and without suicidal ideation. The research that does exist has mainly been conducted within the military. One study found that, among military personnel, lifetime suicidal ideation was associated with unsecure firearm storage (Anestis et al. 2020). Similarly, another study among military members found that service members, who experienced suicidal ideation but did not disclose it, were more likely to store firearms in an unsecure manner (Anestis et al. 2023). While informative, these studies are limited in that they were conducted among military samples and therefore findings may not be generalizable to the civilian population. Further research is needed to determine how suicidal ideation is associated with firearm storage practices among the general population.

Another factor to consider is the perceived risk compared to the benefits of a firearm in the home. Previous research conducted among a sample of firearm owners found that 60% view firearms as increasing home safety, and individuals with such views were more likely to store a firearm loaded and unlocked (Mauri et al. 2019). Additionally, only 3.7% of firearm owners (Mauri et al. 2019) and 6.3% of Veterans believe firearms are associated with risk (Simonetti et al. 2019). These findings suggest that the belief that firearms contribute to safety may be associated with unsecure firearm storage habits. Little is known about the individuals that believe firearms increase risk and how this may impact storage habits. Research should seek to examine how perceived risk and benefits of firearms are impacted by suicidal ideation and how that may also impact firearm storage habits.

Firearm suicide decedents rarely interact with mental health care prior to their death (Bond et al. 2022a), making it difficult to identify and intervene with those at risk on an individual level. Therefore, developing an understanding of how firearm owners who endorse suicidal ideation—general or active—differ from firearm owners who do not report suicidal ideation and nonfirearm owners who do and do not report ideation may facilitate better public health interventions by providing a blueprint that can help identify subgroups of firearm owners at increased risk for suicide. Some research has demonstrated that firearm owners at elevated risk for suicide are less likely to endorse suicidal ideation on traditional measures but tend to still exhibit elevated scores on suicide cognitions (Bryan et al. 2022b). Furthermore, suicide cognitions have been shown to predict suicidal behavior in the absence of suicidal ideation (Bryan et al. 2022c). As such, it is vital that research also considers whether results specific to suicidal ideation are similar when considering suicide cognitions. The present study seeks to address these gaps by utilizing a nationally representative sample to characterize how individuals with firearm access and suicidal ideation (general ideation or active ideation) differ from individuals with firearm access without ideation and individuals without firearm access with and without ideation across multiple domains: demographic characteristics, perception of the risks and benefits of firearm ownership, and the propensity to store firearms loaded and unlocked. Additionally, we seek to further this line of inquiry by examining if suicide cognitions are associated with storage tendencies among individuals with household firearm access. Findings from this study will provide insight into how firearm owners with suicidal ideation differ from other subgroups, which can inform and help tailor public health interventions aimed at reducing suicide risk.

## 2 Method

### 2.1 Participants and Procedures

A nationally representative sample of adults (*n* = 8009) was recruited between May 15 and May 28, 2024 via Ipsos using their probability‐based KnowledgePanel (KP). A total of 12,822 adults were sent the survey, with 8647 (67%) engaging with the informed consent document and 8009 of those individuals (97%) consenting and ultimately being included in the final sample. All procedures were approved by the Rutgers Biomedical and Health Sciences institutional review board, and all participants provided informed consent prior to their participation.

Data weights were computed to optimize representativeness and reflect selection probabilities. Design weights were raked to geodemographic distributions based on the March 2023 supplement of the Current Population Survey and Pew's 2023 National Public Opinion Reference Survey.

### 2.2 Measures

#### 2.2.1 Demographic Characteristics

Sex and age are included in KP member profiles and were not assessed directly by the study team. Political beliefs were assessed by asking participants “How would you characterize your political beliefs?” Answer choices included “highly conservative,” “somewhat conservative,” “moderate,” “somewhat liberal,” and “highly liberal.” Population density was computed by leveraging participant ZIP code, included within KP profile, and matching that information to population density data housed at the National Historical Geographic Information System.

#### 2.2.2 Perceived Value and Risk of Home Firearm Access

Each participant was presented with a set of two items with the instructions “Please select the answer that you believe best reflects the truth.” For both items, the answer choices began with the phrase “Keeping a firearm in or around the home…” For the first item, which assessed perceived protective value of home firearm access during a home invasion, answer choices included “is extremely helpful in protecting people during a home invasion,” “is somewhat helpful in protecting people during a home invasion,” “has no impact on whether people are protected during a home invasion,” “somewhat increases the risk of harm during a home invasion,” and “dramatically increases the risk of harm during a home invasion.” For the second item, which assessed the perceived impact of home firearm access on suicide risk, the answer choices included “is extremely helpful in preventing suicide,” “is somewhat helpful in preventing suicide,” “has no impact on the risk for suicide,” “somewhat increases the risk of suicide,” and “dramatically increases the risk of suicide.” For each variable, scores ranged from 0 to 4.

#### 2.2.3 Firearm Access and Storage

Participants were asked “Is there typically a firearm or firearms stored in or around your home?” Those who endorsed “yes” were categorized as having firearm access. To assess storage, those with firearm access were asked “Are any of the firearms in your home typically stored both unlocked and loaded?”

#### 2.2.4 Suicidal Ideation

Lifetime suicidal ideation was assessed via the Self‐Injurious Thoughts and Behaviors Interview, short report, brief version (SITBI (Nock et al. 2007)). Participants were presented with a list of eight forms of suicidal thoughts, and endorsement of one or more of these thoughts resulted in an individual being categorized as having experienced suicidal ideation. Additionally, four items previously shown (Wastler et al. 2023) to load on an active (vs passive) suicidal ideation factor were utilized to categorize individuals as having or not having experienced lifetime active suicidal ideation.

#### 2.2.5 Suicide Cognitions

Suicide cognitions were assessed using the Brief Suicide Cognitions Scale (B‐SCS (Rudd and Bryan 2021)). Participants were asked to endorse the extent to which six different suicide‐relevant thoughts reflect their current feelings, with answers for each item ranging from 1 (strongly disagree) to 5 (strongly agree) and higher scores indicating more severe levels of suicide cognitions.

### 2.3 Data Analytic Plan

To examine multivariate correlates of loaded and unlocked firearm storage, we utilized three binary logistic regressions—one that considered a lifetime history of any suicidal ideation, one that considered only a lifetime history of active suicidal ideation, and one that considered current severity of suicide cognitions. Firearm access and ideation group (yes/no firearm access, yes/no history of suicidal ideation) served as the independent variable in the first two regressions, and suicide cognitions served as the independent variable in the third. Loaded and unlocked firearm storage served as the dependent variable in all three regressions. Covariates included political beliefs, sex, age, population density, and the degree to which individuals believe firearms are helpful in keeping people safe during a home invasion. Odds ratios served as the index of effect size. To examine differences in the perceived impact of home firearm access on suicide risk, we utilized two multivariate analyses of covariance (MANCOVA), one that considered a lifetime history of any suicidal ideation and one that considered only a lifetime history of active suicidal ideation. Because no agreed‐upon cut point exists for suicide cognitions, we did not utilize that construct as an independent variable in these analyses. In each MANCOVA, the degree to which individuals believe household firearm access increases suicide risk served as the dependent variable, and partial eta‐squared served as the effect size. The list of covariates was identical to the list for the logistic regressions.

## 3 Results

### 3.1 Sample Descriptives

Sample demographics broken down by firearm access status and suicidal ideation (any ideation, active suicidal ideation) status can be found in Table 1. The percentage of individuals of varying characteristics that typically store at least one firearm loaded and unlocked, broken down by suicidal ideation status, can be found in Table 2.

**TABLE 1:** Sample characteristics.

	No firearm access			Firearm access		
	No ideation	Any ideation	Active ideation	No ideation	Any ideation	Active ideation
	% (*n*)	% (*n*)	% (*n*)	% (*n*)	% (*n*)	% (*n*)
Political beliefs						
Highly conservative	7.4 (263)	4.5 (51)	4.1 (20)	17.3 (420)	10.9 (60)	8.9 (19)
Somewhat conservative	17.3 (612)	11.8 (134)	9.0 (44)	30.5 (741)	23.4 (129)	23.6 (50)
Moderate	45.6 (1615)	40.3 (457)	37.2 (182)	38.8 (944)	37.9 (209)	34.4 (73)
Somewhat liberal	19.7 (696)	24.1 (274)	24.0 (117)	18.2 (237)	17.6 (97)	19.3 (41)
Highly liberal	9.9 (352)	19.3 (219)	24.8 (120)	3.7 (90)	10.3 (57)	13.9 (30)
Sex						
Male	49.5 (1785)	41.7 (481)	43.1 (211)	53.9 (1320)	40.0 (222)	44.0 (94)
Female	50.5 (1823)	58.3 (672)	56.9 (278)	46.1 (1129)	60.0 (333)	56.0 (119)
Age						
18–29	18.0 (651)	34.7 (400)	38.2 (187)	13.7 (336)	31.5 (175)	34.2 (73)
30–44	25.1 (906)	33.4 (386)	36.0 (176)	22.2 (544)	32.3 (179)	35.8 (76)
45–59	22.8 (824)	17.8 (205)	15.8 (77)	27.1 (664)	21.8 (121)	20.2 (43)
60+	34.0 (1226)	14.1 (163)	9.9 (49)	37.0 (906)	14.1 (80)	9.8 (21)
Protective value of home firearm Access during home invasion						
Extremely helpful for keeping People safe	18.1 (633)	20.8 (238)	23.9 (116)	55.5 (1354)	47.6 (263)	44.6 (94)
Somewhat helpful for keeping People safe	29.4 (1030)	28.8 (330)	26.8 (131)	31.0 (757)	35.0 (193)	40.8 (86)
No impact on keeping people safe	15.2 (532)	13.5 (154)	14.2 (69)	6.5 (159)	6.0 (33)	5.2 (11)
Somewhat increases risk of harm	16.8 (589)	15.7 (180)	13.2 (65)	4.8 (116)	7.2 (40)	6.4 (13)
Dramatically increases tisk of harm	20.5 (720)	21.2 (242)	21.9 (107)	2.2 (53)	4.2 (23)	3.0 (6)
Perceived impact of home firearm access on suicide risk						
Extremely helpful in preventing suicide	1.4 (50)	0.9 (10)	0.5 (2)	3.1 (75)	2.0 (11)	1.6 (3)
Somewhat helpful in preventing suicide	1.9 (66)	1.3 (15)	1.4 (7)	1.3 (31)	2.0 (11)	0.4 (1)
No impact on suicide risk	28.4 (996)	16.8 (192)	14.3 (69)	56.2 (1356)	35.1 (194)	30.8 (65)
Somewhat increases risk of suicide	31.1 (1090)	30.9 (354)	27.3 (133)	28.6 (691)	39.9 (220)	45.5 (96)
Dramatically increases risk of suicide	37.1 (1299)	50.2 (575)	56.6 (275)	10.8 (261)	21.0 (116)	21.8 (46)

**TABLE 2:** Percentage of individuals of varying characteristics that typically store at least one firearm both loaded and unlocked, sorted by firearm owners with and without a history of suicidal ideation.

	Full firearm access sample	No ideation	Any ideation	Active ideation
	% (*n*)	% (*n*)	% (*n*)	% (*n*)
Political beliefs				
Highly conservative	37.6 (178)	38.2 (159)	33.3 (19)	36.8 (7)
Somewhat conservative	31.4 (268)	31.1 (226)	33.9 (43)	31.3 (15)
Moderate	28.0 (322)	27.8 (259)	28.7 (60)	30.1 (22)
Somewhat liberal	21.6 (71)	19.2 (45)	28.0 (26)	35.0 (14)
Highly liberal	23.1 (34)	20.0 (18)	29.3 (17)	27.6 (8)
Sex				
Male	35.6 (545)	36.2 (473)	32.4 (71)	32.2 (29)
Female	23.3 (334)	21.5 (237)	28.6 (94)	30.3 (36)
Age				
18–29	26.8 (135)	24.9 (82)	30.6 (53)	25.0 (18)
30–44	25.4 (182)	24.4 (132)	27.7 (48)	35.6 (26)
45–59	30.9 (240)	30.3 (198)	33.1 (40)	32.6 (14)
60+	33.2 (323)	33.6 (298)	30.0 (24)	33.3 (7)
Protective value of home firearm access during home invasion				
Extremely helpful for keeping people safe	37.9 (603)	38.1 (506)	36.5 (95)	36.2 (34)
Somewhat helpful for keeping people safe	22.7 (213)	21.6 (162)	26.7 (50)	26.8 (22)
No impact on keeping people safe	16.7 (32)	14.0 (22)	30.3 (10)	36.4 (4)
Somewhat increases risk of harm	10.8 (17)	8.6 (10)	12.8 (5)	28.6 (4)
Dramatically increases risk of harm	17.8 (13)	18.0 (9)	17.4 (4)	16.7 (1)
Perceived impact of home firearm access on suicide risk				
Extremely helpful in preventing suicide	50.0 (41)	50.7 (36)	45.5 (5)	33.3 (1)
Somewhat helpful in preventing suicide	28.6 (12)	19.4 (6)	50.0 (5)	0.0 (0)
No impact on suicide risk	33.7 (517)	34.3 (457)	29.8 (57)	32.8 (20)
Somewhat increases risk of suicide	24.2 (218)	22.7 (156)	28.8 (62)	32.3 (31)
Dramatically increases risk of suicide	22.0 (83)	18.5 (48)	29.3 (34)	26.1 (12)

### 3.2 Correlates of Loaded and Unlocked Firearm Storage

When covarying for sex, age, political beliefs, population density, and the perceived protective value of home firearm access during a home invasion, those with a history of any suicidal ideation (active or passive) had 45% greater odds of endorsing storing firearms loaded and unlocked (Wald = 10.58; *p* = 0.001; OR = 1.45; 95% CI: 1.16–1.80; Table 3). When considering only active suicidal ideation, again those with a history of ideation were more likely to endorse typically storing at least one firearm loaded and unlocked (Wald = 4.99; *p* = 0.025; OR = 1.45; 95% CI: 1.05–2.00). Last, when considering current suicide cognitions rather than lifetime suicidal ideation, a similar pattern emerged, with individuals exhibiting 4% greater odds of endorsing typically storing at least one firearm loaded and unlocked for every unit increase in total suicide cognition score (Wald = 12.78, *p* < 0.001; OR = 1.04; 95% CI: 1.02–1.07).

**TABLE 3:** Binary logistic regressions examining the likelihood of typically storing at least one firearm both loaded and unlocked among those with firearm access with and without suicidal ideation (any suicidal ideation and active suicidal ideation) and among all adults with household firearm access.

	Wald	*p*	OR	95% CI
Any suicidal ideation (lifetime)				
Political beliefs	1.52	0.217	0.95	0.87–1.03
Sex (ref: male)	37.55	< 0.001	0.59	0.50–0.70
Age (ref: 18–29)	28.50	< 0.001		
30–44	0.02	0.886	0.98	0.75–1.28
45–59	4.16	0.041	1.31	1.01–1.71
60+	16.84	< 0.001	1.72	1.33–2.22
Population density	0.68	0.411	1.00	1.00–1.00
Protective value of firearm access during home invasion	77.00	< 0.001	0.60	0.54–0.68
Firearm access and any ideation (ref: firearm access and no suicidal ideation)	10.58	0.001	1.45	1.16–1.80
Active suicidal ideation (lifetime)				
Political beliefs	1.20	0.273	0.95	0.88–1.04
Sex (ref: male)	34.97	< 0.001	0.60	0.51–0.71
Age (ref: 18–29)	25.44	< 0.001		
30–44	0.10	0.748	0.96	0.73–1.25
45–59	3.13	0.077	1.26	0.98–1.64
60+	14.19	< 0.001	1.63	1.26–2.10
Population density	0.66	0.417	1.00	1.00–1.00
Protective value of firearm access during home invasion	75.60	< 0.001	0.61	0.54–0.69
Firearm access and active ideation (ref: firearm access and no active ideation)	4.99	0.025	1.45	1.05–2.00
All adults with household firearm access				
Political beliefs	1.15	0.283	0.95	0.88–1.04
Sex (ref: male)	34.50	< 0.001	0.60	0.51–0.72
Age (ref: 18–29)	29.88	< 0.001		
30–44	0.02	0.899	1.02	0.78–1.33
45–59	4.80	0.028	1.34	1.03–1.75
60+	18.87	< 0.001	1.77	1.37–2.29
Population density	0.69	0.405	1.00	1.00–1.00
Protective value of firearm access during home invasion	71.85	< 0.001	0.62	0.55–0.69
Suicide cognitions	12.78	< 0.001	1.04	1.02–1.07

*Note:* Protective value of home firearm access is scored such that lower scores indicate greater perceived protective value during a home invasion.

In each analysis, sex differences emerged, with women exhibiting lower odds than men of typically storing at least one firearm loaded and unlocked. As a post hoc examination of the impact of sex on our findings, we chose to re‐run our analyses, first only including males with household firearm access and then again only including females with household firearm access. Among men (Table 4), neither the results for any suicidal ideation (Wald = 0.36; *p* = 0.548; OR = 1.11; 95% CI: 0.80–1.53) nor the results for active suicidal ideation (Wald = 0.01; *p* = 0.928; OR = 1.02; 95% CI: 0.63–1.65) were significant. Results for suicide cognitions, however (Wald = 3.97; *p* = 0.046; OR = 1.03; 95% CI: 1.00–1.07), remained significant. Among women (Table 5), results for any suicidal ideation (Wald = 14.42; *p* < 0.001; OR = 1.80; 95% CI: 1.33–2.44), active suicidal ideation (Wald = 8.05; *p* = 0.005; OR = 1.89; 95% CI: 1.22–2.94), and suicide cognitions (Wald = 8.66; *p* = 0.003; OR = 1.05; 95% CI: 1.02–1.08) were significant and in the same direction as the full sample of individuals with household firearm access.

**TABLE 4:** Binary logistic regressions examining the likelihood of typically storing at least one firearm both loaded and unlocked among men with firearm access with and without suicidal ideation (any suicidal ideation and active suicidal ideation) and among all men with household firearm access.

	Wald	*p*	OR	95% CI
Any suicidal ideation (lifetime)				
Political beliefs	0.67	0.414	0.95	0.85–1.07
Age (ref: 18–29)	21.17	< 0.001		
30–44	0.02	0.877	1.03	0.72–1.47
45–59	4.66	0.031	1.46	1.04–2.05
60+	13.90	< 0.001	1.89	1.35–2.64
Population density	0.95	0.331	1.00	1.00–1.00
Protective value of firearm access during home invasion	49.73	< 0.001	0.55	0.47–0.65
Firearm access and any ideation (ref: firearm access and no suicidal ideation)	0.36	0.548	1.11	0.80–1.53
Active suicidal ideation (lifetime)				
Political beliefs	0.61	0.435	0.96	0.85–1.07
Age (ref: 18–29)	20.75	< 0.001		
30–44	0.01	0.911	1.02	0.71–1.46
45–59	4.40	0.036	1.44	1.02–2.02
60+	13.49	< 0.001	1.85	1.33–2.58
Population density	0.96	0.328	1.00	1.00–1.00
Protective value of firearm access during home invasion	49.49	< 0.001	0.55	0.47–0.65
Firearm access and active ideation (ref: firearm access and no active ideation)	0.01	0.928	1.02	0.63–1.65
All adults with household firearm access				
Political beliefs	0.75	0.388	0.95	0.85–1.07
Age (ref: 18–29)	25.02	< 0.001		
30–44	0.24	0.628	1.09	0.76–1.57
45–59	6.10	0.014	1.55	1.09–2.18
60+	17.66	< 0.001	2.06	1.47–2.89
Population density	1.03	0.311	1.00	1.00–1.00
Protective value of firearm access during home invasion	47.98	< 0.001	0.56	0.47–0.66
Suicide cognitions	3.97	0.046	1.03	1.00–1.07

*Note:* Protective value of home firearm access is scored such that lower scores indicate greater perceived protective value during a home invasion.

**TABLE 5:** Binary logistic regressions examining the likelihood of typically storing at least one firearm both loaded and unlocked among women with firearm access with and without suicidal ideation (any suicidal ideation and active suicidal ideation) and among all women with household firearm access.

	Wald	*p*	OR	95% CI
Any suicidal ideation (lifetime)				
Political beliefs	1.08	0.299	0.93	0.82–1.06
Age (ref: 18–29)	8.13	0.043		
30–44	0.22	0.637	0.91	0.60–1.36
45–59	0.29	0.587	1.12	0.75–1.68
60+	3.57	0.059	1.47	0.99–2.21
Population density	0.01	0.925	1.00	1.00–1.00
Protective value of firearm access during home invasion	27.33	< 0.001	0.66	0.56–0.77
Firearm access and any ideation (ref: firearm access and no suicidal ideation)	14.42	< 0.001	1.80	1.33–2.44
Active suicidal ideation (lifetime)				
Political beliefs	0.88	0.347	0.94	0.83–1.07
Age (ref: 18–29)	6.60	0.090		
30–44	0.40	0.527	0.88	0.59–1.32
45–59	0.10	0.755	1.07	0.71–1.60
60+	2.32	0.128	1.36	0.92–2.02
Population density	0.00	0.967	1.00	1.00–1.00
Protective value of firearm access during home invasion	26.32	< 0.001	0.66	0.57–0.78
Firearm access and active ideation (ref: firearm access and no active ideation)	8.05	0.005	1.89	1.22–2.94
All adults with household firearm access				
Political beliefs	0.42	0.519	0.96	0.84–1.09
Age (ref: 18–29)	6.94	0.074		
30–44	0.17	0.676	0.92	0.61–1.38
45–59	0.19	0.663	1.09	0.73–1.64
60+	3.03	0.082	1.43	0.96–2.13
Population density	0.00	0.980	1.00	1.00–1.00
Protective value of firearm access during home invasion	24.49	< 0.001	0.68	0.58–0.79
suicide cognitions	8.66	0.003	1.05	1.02–1.08

*Note:* Protective value of home firearm access is scored such that lower scores indicate greater perceived protective value during a home invasion.

### 3.3 Perceived Protective Value and Suicide Risk of Home Firearm Access

Results from the MANCOVA examining any suicidal ideation (see Table 6) indicated that the four groups differed in their perception of the degree to which home firearm access increases the risk of suicide (F = 72.64; *p* < 0.001; _p_η^2^ = 0.03). Those with no firearm access and a history of suicidal ideation endorsed a greater belief that home firearm access increases risk for suicide (EMM = 4.11; SE = 0.02) relative to those with no firearm access and no history of suicidal ideation (EMM = 3.90; SE = 0.01) and those with firearm access and a history of suicidal ideation (EMM 3.90; SE = 0.03), with the latter two groups also differing from those with firearm access and no history of suicidal ideation (EMM = 3.68; SE = 0.02)^1^. Similarly, results from the MANCOVA examining active suicidal ideation (see Table 6) indicated that the four groups differed in their perception of the degree to which home firearm access increases the risk of suicide (F = 40.55; *p* < 0.001; _p_η^2^ = 0.02). Those with no firearm access and a history of active suicidal ideation endorsed a greater belief that home firearm access increases risk for suicide (EMM = 4.19; SE = 0.04) relative to those with no firearm access and no history of active suicidal ideation (EMM = 3.92; SE = 0.01) and those with firearm access and a history of active suicidal ideation (EMM 3.96; SE = 0.05), with the latter two groups also differing from those with firearm access and no history of active suicidal ideation (EMM = 3.70; SE = 0.02).

**TABLE 6:** Belief in the degree to which firearm access increases risk for suicide across groups (any suicidal ideation and active suicidal ideation).

Any suicidal ideation (lifetime)	*F*	*p*	_p_η^2^
Political beliefs	389.30	< 0.001	0.05
Sex	1.63	0.201	0.00
Age	0.82	0.364	0.00
Population density	−0.38	0.539	0.00
Protective value of firearm access during home invasion	687.36	< 0.001	0.08
Firearm access and suicidal ideation groups	72.64	< 0.001	0.03
	N	EMM	SE
No firearm access—no suicidal ideation	3734	3.90_a_	0.01
No firearm access—suicidal ideation	1172	4.11_b_	0.02
Firearm access—no suicidal ideation	2638	3.68_c_	0.02
Firearm access—suicidal ideation	578	3.90_a_	0.03

Active suicidal ideation (lifetime)	*F*	*p*	_p_η^2^
Political beliefs	394.40	< 0.001	0.05
Sex	0.53	0.466	0.00
Age	0.01	0.910	0.00
Population density	0.41	0.422	0.00
Protective value of firearm access during home invasion	436.82	< 0.001	0.08
Firearm access and suicidal ideation groups	40.55	< 0.001	0.02
	N	EMM	SE
No firearm access—no active suicidal ideation	4415	3.92_a_	0.01
No firearm access—active suicidal ideation	491	4.19_b_	0.04
Firearm access—no active suicidal ideation	2991	3.70_c_	0.02
Firearm access—active suicidal ideation	225	3.96_a_	0.05

*Note:* Higher scores indicate a belief that firearm access increases the risk of suicide. Perceived impact of household firearm access on suicide risk was scored as follows: 1 = Keeping a firearm in or around the home is extremely helpful in preventing suicide, 2 = Keeping a firearm in or around the home is somewhat helpful in preventing suicide, 3 = Keeping a firearm in or around the home has no impact on the risk for suicide, 4 = Keeping a firearm in or around the home somewhat increases the risk of suicide, and 5 = Keeping a firearm in or around the home dramatically increases the risk of suicide. Distribution of scores on this measure was as follows: Full sample (m = 3.84, SD = 0.93; Min = 1, Max—5); those with firearm access (m = 3.49; SD = 0.84; Min = 1, Max = 5); loaded and unlocked (m = 3.33, SD = 0.85, Min = 1, Max = 5); not loaded and unlocked (m = 3.56, SD = 0.83, Min = 1, Max = 5). Significant a,b and c represents *p* < .05 value.

## 4 Discussion

The primary aim of this study was to examine differences in firearm storage and perceptions of the impact of home firearm access on suicide risk across groups of individuals who vary with respect to both firearm access and lifetime history of suicidal thoughts (general ideation or active ideation) or current suicide cognitions. Suicide prevention efforts have historically been hampered by the tendency for firearm suicide decedents to have avoided mental health care and thus eluded detection as high risk prior to death (Bond et al. 2022a). As such, the importance of understanding if and how those with firearm access and a history of suicidal thoughts differ from others with firearm access and from those with suicidal ideation but no firearm access is clear: without a sense of the behaviors and perceptions of this group, suicide prevention will continue to struggle with the same obstacles. Our data facilitated analyses that can characterize national trends and our findings highlighted a series of meaningful differences between groups that may aid in our efforts to properly characterize firearm owners with the propensity to experience suicidal ideation.

Our findings highlight several concerning points. At the univariate level, those with a history of suicidal ideation typically endorsed that household firearm access is associated with elevated suicide risk. For instance, the majority of those with a history of ideation (56.6% with lifetime active ideation; 50.2% with lifetime ideation more generally) but no firearm access indicated that household firearm access dramatically increases suicide risk (vs 37.1% with no history of ideation). Findings were similar, though more muted, among those with firearm access (21.8% with lifetime active ideation and 21.0% with lifetime ideation more generally vs. 10.8% with no history of ideation). Similarly, among those with firearm access and who believe household firearm access dramatically increases suicide risk, 26.1% of those with lifetime active ideation and 29.3% of those with lifetime ideation more generally (vs 18.5% of those with no history of ideation) reported typically storing at least one firearm both loaded and unlocked. This pattern was reinforced in our multivariate analysis, which covaried for other relevant factors (e.g., perceived protective value of firearms during home invasion, sex). Here again, those with a history of suicidal ideation (active or more general) had 45% higher odds of endorsing loaded and unlocked firearm storage. Similarly, when we considered current suicide cognitions rather than lifetime suicidal ideation, the results indicated that those with more severe levels of suicide cognitions had higher odds of loaded and unlocked firearm storage.

Our findings align well with relevant prior research. For instance, Bryan and colleagues (Bryan et al. 2019) reported that, among firearm‐owning military service members and Veterans in primary care, those with a history of suicidal thoughts were more likely to store firearms loaded and unlocked. Anestis and colleagues (Anestis et al. 2020) replicated that finding in a large sample of National Guardsmen. In each of these prior instances, the findings were specific to those with military experience. Our findings thus advance beyond prior work by providing a nationally representative perspective that optimizes the generalizability of the results. Our findings also extend beyond prior related work by covarying not only for demographic factors but also for community‐level risk (population density) and factors that weigh heavily into the decision to own a firearm and how to store that firearm (perceptions of protective value during a home invasion).

Our post hoc follow‐up analyses indicated that the results for unsecure firearm storage were significant among women, but largely non‐significant among men. Women are less likely than men to identify as firearm owners; however, sex differences are less robust with respect to household firearm access. Our findings indicate that women with a history of suicidal thoughts—whether active or more general—or current suicide cognitions are prone to living in homes with firearms stored loaded and unlocked. For some, this may reflect a decision on their part to make a firearm more readily accessible in response to their suicidal thoughts. For others, it might reflect that women prone to suicidal thoughts are more likely to live with partners who make riskier decisions about firearm storage and that such storage decisions are not directly related to the suicidal ideation. In this case, our findings would be more indicative of environmental risk more common among women with ideation among firearm owning homes than they would be about risky firearm storage decisions among women with ideation.

Although our findings align with prior work (Bryan et al. 2019), thus enhancing confidence in their generalizability across communities, it remains unknown precisely why those with a more extensive history of suicidal thoughts—and those with more severe current suicide cognitions—tend to be more likely to store firearms unsecured, at least among women. One concerning possibility is that such storage decisions are driven specifically by suicidal ideation. Indeed, in addition to our concerning findings related to firearm storage practices, we also found that individuals with firearm access and a history of suicidal thoughts reported a belief in a particularly strong link between firearm access and suicide risk, indicating that these individuals understand the risks involved in firearm access and, despite their own experiences with suicidal thinking, nonetheless choose not only to keep a firearm in and around the home, but often to store it loaded and unlocked. This could mean that some individuals are intentionally maintaining an environment that is conducive to suicide risk as a means of preparing for a suicide attempt. Whether or not such behavior would constitute active planning is open to question; however, future research that more directly assesses whether storage behavior is motivated by or even used as a means of regulating suicidal thoughts would have substantial clinical value.

An alternative and more benign interpretation might simply be that suicidal thoughts are more common among individuals with a history of risk‐taking behavior (Ammerman et al. 2016). In this sense, it may be that suicidal ideation or cognitions co‐occur with unsecure firearm storage, but that the two phenomena are not directly related. Rather, the same risk factors that increased the odds of an individual experiencing suicidal thoughts may have increased their odds of acquiring a firearm and storing it loaded and unlocked. In this case, the importance of policies and programs that encourage safer methods of home protection (e.g., home security systems) would be vital, as they may lower the odds that individuals less averse to risk choose dangerous methods for home protection (e.g., unsecured firearms) that increase their baseline risk for suicide.

Several limitations of this study are worth noting. First, our findings were cross‐sectional and self‐report, which introduces potential response biases and precludes any tests of causality. Second, our results focused on firearm owners with lifetime ideation rather than firearm owners acutely experiencing suicidal thoughts. Although we had access to information on past month suicidal thoughts, sample size limitations prevented a thorough analysis of those results and, furthermore, the dynamic nature of suicidal thoughts renders even assessments of past month suicidal thoughts of limited relevance to the experiences of the current and subsequent moments (Kleiman et al. 2017). Our findings thus may speak less about which firearm owners are experiencing suicidal thoughts now and more to which firearm owners have a generally elevated risk of suicidal thoughts. Our results are also incapable of identifying specific individuals who do not believe firearms provide any element of safety and who maintain firearm access specifically for the purpose of eventually using them in a suicide attempt. Such individuals who undoubtedly be of particular concern—especially if they are experiencing an acute suicidal crisis—and future research should endeavor to better understand this high‐risk subgroup. Last, the version of the SCS that we utilized included an item that directly asks about suicidal thoughts. Although we do not have any empirical rationale to assume that the SCS results largely reflect suicidal thoughts rather than the cognitions that underlie suicidal thinking, it is worth noting that this item may have had some influence on the pattern of findings for those analyses.

## 5 Public Health Implications

Our findings highlight that elevated perceptions of the utility of home firearm access during home invasions may potentially supersede perceptions of risk for suicide when individuals make decisions about home firearm access and storage. In this sense, our findings highlight the importance of population‐level corrections in the general understanding of how firearms influence the potential for various outcomes and what role they historically have served in preventing and facilitating tragedies.

Consistent with this notion, recent research on suicide prevention has generally called for population‐level interventions that avoid the need to identify suicide risk prior to implementing means safety interventions (Anestis et al. 2021a). Our findings do not contradict that call, but they do highlight a path for better characterizing the firearm owners most likely not only to be engaging in dangerous firearm behaviors (loaded and unlocked storage) but also those with a greater propensity for experiencing suicidal thoughts. This provides minimal insight on the individual level but can provide meaningful guidance for larger public health efforts, including messaging, by highlighting specific subgroups to whom messaging might need to be directed in order to increase the odds that those at greatest risk are encountering and internalizing messaging on the importance of secure firearm storage. Research has consistently demonstrated that diverse subgroups of firearm owners consistently report that law enforcement, military service members, and Veterans represent trusted messengers on the issue of secure firearm storage more broadly and specifically with respect to suicide prevention (Anestis et al. 2021b, 2022). Importantly, given our findings related to sex differences, this list of credible messengers remained the same when specifically considering women with firearm access. In reaching such individuals, it will be vital to ensure that the messages are received through credible channels, as prior research has demonstrated that the perceived credibility of messaging related to firearm storage is dependent on the perceived credibility of the source, regardless of the content of the message (Bandel et al. 2022).

## Author Contributions

**Michael D. Anestis:** conceptualization (lead), formal analysis (lead), funding acquisition (lead), methodology (lead), supervision (lead), writing – original draft (lead), writing – review and editing (lead). **Allison E. Bond:** writing – original draft (supporting), writing – review and editing (equal).

## Conflicts of Interest

The authors declare no conflicts of interest.

## Data Availability

Data can be made available upon request.

## Endnotes

^1^
For comparison, we also examined if these groups differed in the perceived protective value of home firearm access during home invasion. Here again, the four groups differed (*F* = 312.33; *p* < 0.001; _p_η^2^ = 0.10). Both those with firearm access and no history of suicidal ideation (EMM = 0.98, SE = 0.02) and those with firearm access and suicidal ideation (EMM = 1.00; SE = 0.05) perceived greater protective value than did those with no firearm access and suicidal ideation (EMM = 1.70; SE = 0.03), with the latter group also perceiving greater protective value than those with no firearm access and no history of suicidal ideation (EMM = 1.85; SE = 0.02).
